# Effects of dual-task training on health-related quality of life and cognitive function in people with Parkinson’s disease: a systematic review and meta-analysis

**DOI:** 10.3389/fnagi.2026.1939266

**Published:** 2026-09-28

**Authors:** Leandro Cerda-Pérez, Catalina Gutiérrez-Rojas, Valentina Retamal-Salazar, Karina Rodríguez-Faúndez, Javiera Venegas-Alarcón, Jordan Hernandez-Martinez, Cristian Sandoval-Vásquez, Pablo Valdés-Badilla, Edgar Vásquez-Carrasco

**Affiliations:** 1 School of Occupational Therapy, Faculty of Psychology, Universidad de Talca, Talca, Chile; 2 Department of Physical Activity Sciences, Universidad de Los Lagos, Osorno, Chile; 3 Department of Education, Faculty of Humanities, Universidad de La Serena, La Serena, Chile; 4 Carrera de Terapia Ocupacional, Facultad de Ciencias de la Salud, Universidad Autónoma de Chile, Temuco, Chile; 5 Departamento de Medicina Interna, Facultad de Medicina, Universidad de La Frontera, Temuco, Chile; 6 Department of Physical Activity Sciences, Faculty of Education Sciences, Universidad Católica del Maule, Talca, Chile; 7 Sports Coach Career, School of Education, Universidad Viña del Mar, Viña del Mar, Chile; 8 Centro de Investigación en Ciencias Cognitivas, Faculty of Psychology, Universidad de Talca, Talca, Chile; 9 VITALIS Longevity Center, Universidad de Talca, Talca, Chile

**Keywords:** adults, aged, interventions, neurodegenerative disorders, neurological rehabilitation

## Abstract

**Introduction:**

Dual-task training is proposed as a rehabilitative strategy to mitigate motor and cognitive impairments in patients with Parkinson’s disease (PD). This systematic review and meta-analysis sought to consolidate existing information regarding the impact of dual-task therapies on health-related quality of life (HRQoL) and cognitive performance in middle-aged and older persons diagnosed with PD.

**Methods:**

A systematic review of randomized controlled trials (RCTs) published to May 2026 was performed utilizing PubMed, EBSCOhost, Scopus, and Web of Science. The chosen studies adhered to the PICOS framework. The Methodological quality and certainty of evidence were assessed using the Cochrane Risk of Bias 2 tool and GRADE framework, respectively. The review protocol is recorded in PROSPERO under the number CRD420251159680.

**Results:**

Out of 3,037 identified records, 10 RCTs met the eligibility criteria and were included in the systematic review, of which 9 contributed data to the meta-analysis. The meta-analysis showed a statistically significant but modest effect on HRQoL measured with the Parkinson’s Disease Questionnaire-39 (SMD = 0.35; 95% CI: 0.03–0.67; *p =* 0.03). No significant between-group effects were observed for TMT-A (MD = 0.99; 95% CI: -3.38 to 5.37; *p =* 0.66), TMT-B (SMD = 0.02; 95% CI: -0.24 to 0.29; *p =* 0.86), or MoCA (SMD = 0.19; 95% CI: -0.26 to 0.64; *p =* 0.41).

**Conclusion:**

Dual-task interventions may produce a modest improvement in HRQoL in people with Parkinson’s disease, whereas the current evidence does not demonstrate significant benefits for the cognitive outcomes assessed. These findings should be interpreted cautiously given the limited number of studies, methodological limitations, and clinical heterogeneity across interventions.

**Systematic review registration:**

https://www.crd.york.ac.uk/PROSPERO/view/CRD420251159680, identifier CRD420251159680.

## Introduction

Parkinson’s disease (PD) is the second most common neurodegenerative disorder; it affects about 1-2% of people aged 65 years and older (Ou et al., 2021). The prevalence and years lived with disability due to PD have increased substantially over recent decades, making it one of the world’s fastest-growing neurological diseases and a major public health challenge (Chaudhary et al., 2025; Ou et al., 2021). Although motor symptoms are the main clinical manifestations of PD, non-motor symptoms are also highly relevant and frequently underestimated. This underrecognition may be related to limited patient awareness and reluctance to disclose symptoms, with approximately 62% of patients not reporting non-motor concerns to healthcare providers (Hurt et al., 2019; Marinova and Danovska, 2020). Systematic reviews show that cognitive impairment, mood disorders, fatigue, and sleep problems occur across all stages of PD and substantially worsen disease burden by limiting everyday functioning (Cong et al., 2022; Ernst et al., 2023; Peña-Zelayeta et al., 2025).

Both motor and non-motor symptoms exert a significant negative impact on health-related quality of life (HRQoL), a multidimensional construct reflecting individuals’ perceptions of physical functioning, emotional well-being, social participation, and independence in daily activities (Candel-Parra et al., 2021; Cegarra et al., 2023). Importantly, HRQoL impairments extend beyond the individual with PD, affecting family members and caregivers by increasing care demands and restricting social engagement. These broader consequences underscore the need for therapeutic approaches that concurrently target motor and cognitive dysfunctions to achieve meaningful, patient-centered outcomes (Gawroñska et al., 2025; Magano et al., 2026).

The management of PD relies on a comprehensive and multidisciplinary framework encompassing pharmacological, surgical, and rehabilitative interventions (Foltynie et al., 2024; Goldman et al., 2024). Although pharmacological treatments remain central to symptom control, long-term use is often accompanied by adverse effects and motor complications. Surgical interventions, while effective for selected patients, are costly and not universally accessible. These limitations have prompted increasing interest in non-pharmacological rehabilitation strategies as complementary approaches to standard care (Muleiro Alvarez et al., 2024).

Within this context, dual-task training (DT) has gained attention as a rehabilitation approach. In the present review, direct cognitive–motor DT refers to interventions in which an identifiable motor task and an identifiable cognitive task are performed concurrently (Johansson et al., 2023; McIsaac et al., 2015; Vásquez-Carrasco et al., 2025). The cognitive demand may be presented as a separate task or integrated through a cognitive rule that directly determines the motor response (Herold et al., 2018). Because some rehabilitation approaches address dual-task performance within broader multicomponent programs or through adjunctive strategies, these interventions were distinguished from direct DT training when dual-task performance represented an explicit therapeutic target. Accordingly, these approaches were not assumed to be mechanistically equivalent and were characterized separately throughout the review. Direct DT interventions commonly combine gait, balance, treadmill walking, or functional mobility tasks with cognitive demands involving attention, executive function, memory, verbal processing, calculation, decision-making, or response inhibition (Herold et al., 2018; Johansson et al., 2023; Vásquez-Carrasco et al., 2025). This approach differs from purely dual-motor paradigms, in which two motor tasks are performed simultaneously without an explicit cognitive component. Therefore, the primary focus of this review is on interventions targeting cognitive–motor integration rather than dual-motor practice alone.

Dual-task interference is not specific to PD and can also occur in healthy young and older adults, with its magnitude varying according to factors such as task difficulty and task prioritization (O’Shea et al., 2002; Plummer-D’Amato et al., 2012). Although evidence that dual-task interference is consistently greater in people with PD remains inconclusive, it is clinically relevant in this population because many everyday activities require individuals to maintain posture, walk, turn, or perform functional tasks while simultaneously processing information, dividing attention, inhibiting responses, or making decisions (García-López et al., 2023; Kelly et al., 2012). Under these conditions, dual-task interference may contribute to impairments in gait performance, balance, postural control, functional confidence, and participation in daily life (Ali et al., 2022; Zheng et al., 2022). This functional relevance has contributed to increasing interest in rehabilitation approaches that expose individuals to concurrent motor and cognitive demands, with the aim of improving cognitive-motor integration and reducing dual-task interference (Herold et al., 2018). Recent systematic reviews and meta-analyses suggest that DT and other motor–cognitive interventions may improve gait and balance outcomes; however, findings regarding cognitive function and HRQoL remain inconsistent and comparatively less established (Kao et al., 2025; Li et al., 2020). Although recent randomized controlled trials have reported promising cognitive findings, no meta-analysis to date has comprehensively examined the effects of cognitive-motor DT interventions on both cognitive outcomes and HRQoL in people with PD (Durán-Navarrete et al., 2024). Therefore, the present systematic review with meta-analysis aimed to evaluate the effectiveness of dual-task training on health-related quality of life and cognitive function in middle-aged and older adults with Parkinson’s disease.

## 2 Methods

### 2.1 Protocol and registration

This systematic review and meta-analysis adhered to the methodological guidance of the Cochrane Collaboration (Higgins et al., 2023) and is reported in accordance with the Preferred Reporting Items for Systematic Reviews and Meta-Analyses (PRISMA) 2020 guidelines, including the completed checklist and flow diagram (Page et al., 2021). The review protocol was registered in the PROSPERO international database of systematic reviews (CRD420251159680) before the final literature search update and quantitative synthesis.

### 2.2 Eligibility criteria

Eligible studies were peer-reviewed randomized controlled trials (RCTs) published in any language, with no restrictions on publication year, and available up to May 2026. Full-text articles written in languages not understood by the review team were translated using machine-assisted translation, and the translated content was independently checked by a second reviewer before eligibility assessment and data extraction. No study was excluded based on language. To operationalize intervention eligibility, studies were classified according to how the intervention addressed cognitive–motor dual-task performance. Direct DT interventions were eligible when they involved repeated practice of a motor task performed concurrently with an explicit cognitive task or with a cognitive rule incorporated into the motor task. Multicomponent rehabilitation programs were also eligible when an identifiable and repeatedly practiced cognitive–motor or dual-task component formed part of the intervention. In addition, adjunctive DT-targeted interventions were retained when dual-task performance represented an explicit therapeutic target of the trial and repeated motor training was combined with an adjunctive strategy intended to influence processes relevant to dual-task performance. These approaches were distinguished from direct DT training during study characterization and interpretation. Eligibility was therefore determined from the actual intervention content and therapeutic target rather than from the intervention label alone. Studies were excluded if they consisted of conference abstracts, books or book chapters, editorials, letters, study protocols, reviews, case reports, non-randomized studies, or other forms of non-original research. Eligibility was assessed using the PICOS framework (Population, Intervention, Comparator, Outcomes, and Study design), which is detailed in Table 1.

**TABLE 1:** Selection criteria used in the systematic review.

Category	Inclusion	Exclusion
Population	Middle-aged and older adults diagnosed with PD, irrespective of sex. Middle age was defined as a mean age ≥45 years.	Studies including participants whose primary diagnosis was not PD (e.g., multiple sclerosis, traumatic brain injury, amyotrophic lateral sclerosis).
Intervention	Repeated training involving the concurrent performance of a motor task and an explicitly identifiable cognitive task. The cognitive component could be added to the motor task or incorporated into it through a cognitive rule or stimulus that guided the motor response.	Motor-only exercise; dual-motor training without an explicit cognitive component; sequentially delivered motor and cognitive training; general exercise involving attentional demands but no identifiable cognitive task; and interventions in which dual-task performance was assessed but not repeatedly trained.
Comparison	Interventions compared with active or inactive (usual care or no intervention) control groups.	Absence of a comparator group and/or lack of baseline or follow-up data.
Outcomes	At least one validated measure of cognitive function (e.g., TMT, MoCA) and/or HRQoL (e.g., PDQ-39).	Absence of outcome data at baseline and/or follow-up assessments.
Study design	RCTs including pre- and post-intervention assessments.	Non-randomized designs, including controlled clinical trials, retrospective or prospective observational studies, and cross-sectional studies.

HRQoL: Health-related quality of life; MoCA: Montreal cognitive assessment; PD: Parkinson’s disease; PDQ-39: Parkinson’s disease questionnaire–39; RCT: Randomized controlled trial; TMT: Trail making test.

### 2.3 Information and database search process

A systematic literature search was conducted utilizing four main electronic databases: PubMed, EBSCOhost, Scopus, and the Web of Science Core Collection. The search process took place up to May 2026. Terms from the U.S. National Library of Medicine’s MeSH were integrated with relevant free-text keywords related to DT interventions and their effects on HRQoL and cognitive function in middle-aged and older adults with PD. The term “paralysis agitans” is a historical designation for Parkinson’s disease and was included to retrieve older records or publications indexed using legacy terminology.

Search strategies were customized to meet the distinct requirements of each database. The search strategy is provided below: (“dual task” OR “dual-task” OR “dual tasking” OR “dual-task training” OR “dual task training” OR “dual-task intervention” OR “dual task intervention” OR “dual-task exercise” OR “dual task exercise” OR “dual motor task” OR “motor-cognitive” OR “motor cognitive” OR “cognitive-motor”) AND (“Parkinson’s disease” OR “Parkinson disease” OR “Parkinson” OR “PD” OR “Parkinson syndrome” OR “paralysis agitans”) AND (“adult” OR “aged” OR “adults” OR “elderly” OR “older adult” OR “older individuals”). To maximize search sensitivity, the electronic searches were repeated without age-related terms, and eligibility based on participant age was assessed during full-text screening. This additional search identified no additional eligible studies and therefore did not alter the final study sample.

An external expert with a doctoral degree in health-related sciences and extensive experience in peer-reviewed research independently audited the eligibility decisions after completion of the primary screening process. The expert did not participate in the initial title/abstract or full-text screening and served exclusively as an additional methodological cross-check. A final search update was performed in May 2026 to identify potentially relevant new records and to verify whether any corrections, retractions, or updates had been issued for the included studies.

### 2.4 Study selection and data extraction procedures

During full-text screening, intervention eligibility was assessed according to the predefined DT classification described above. Reviewers examined the actual training content and therapeutic target to determine whether interventions involved direct cognitive–motor DT practice, contained an identifiable DT component within a multicomponent program, or represented an adjunctive intervention explicitly targeting dual-task performance. All retrieved references were imported into Mendeley Reference Manager (version 2.116.2), and duplicate records were removed before screening. Four reviewers independently screened every record at the title and abstract stage and subsequently assessed every potentially eligible full-text article. Each reviewer evaluated the same set of records using the predefined eligibility criteria. During both screening stages, study reports were anonymized, and reviewers were blinded to study authors, institutional affiliations, journal titles, and one another’s decisions until all assessments were completed. Disagreements were resolved through consensus. Reasons for exclusion during full-text assessment were systematically recorded. Data extraction was performed using a predefined standardized form developed in Microsoft Excel^®^ (version 2506; Microsoft Corporation, Redmond, WA, USA), following Cochrane methodological guidance (Higgins et al., 2023). The same four reviewers independently extracted data from every included study and remained blinded to one another’s extraction forms until completion. The completed forms were subsequently compared, and discrepancies were resolved through consensus. Two additional reviewers audited the final eligibility decisions and consolidated dataset. The external expert participated exclusively in this auditing process and did not perform the primary screening or data extraction. The extracted variables comprised authorship and country of origin, study design, sample characteristics, group allocation (n), mean participant age, comprehensive descriptions of the intervention and comparator conditions, training volume parameters (frequency, duration, and intensity), assessed cognitive domains, health-related quality of life measures, and primary study outcomes.

### 2.5 Assessment of methodological quality

The level of evidence of the included studies was descriptively classified according to the Oxford Centre for Evidence-Based Medicine framework (Manterola and Zavando, 2009). This classification was applied after study inclusion and was not used as an additional eligibility criterion. Methodological quality was assessed separately using the Cochrane Risk of Bias 2 tool, as described below.

### 2.6 Risk of bias

The risk of bias of the included randomized controlled trials was assessed using the revised Cochrane Risk of Bias tool (RoB 2) (Higgins et al., 2020). Each included study was independently assessed by four reviewers using anonymized reports. Reviewers were blinded to study identifiers and one another’s judgments until all assessments were completed. Disagreements were resolved through consensus, and the final judgments were independently cross-checked by two additional reviewers.

### 2.7 Measures of meta-analysis

The outcomes included in the quantitative synthesis were selected according to the predefined review domains and the availability of sufficiently comparable data across the included trials. HRQoL was represented by the PDQ-39 because it was the disease-specific HRQoL measure most consistently reported across studies and provided sufficient comparable data for quantitative pooling. Cognitive outcomes were synthesized separately according to the assessment instrument. TMT-A was considered primarily as a measure of visual attention and processing speed, TMT-B as a measure with greater demands on executive control and cognitive flexibility, and MoCA as a measure of global cognitive function. These instruments were included in the meta-analysis because they were reported by multiple eligible studies with sufficiently comparable post-intervention data. Other cognitive or HRQoL measures were retained in the qualitative synthesis when quantitative pooling was not considered appropriate because of insufficient reporting or lack of comparability across studies.

Effect estimates with 95% confidence intervals (CIs) were calculated using Review Manager software (RevMan 5.4, The Cochrane Collaboration, London, UK). Standardized mean differences (SMDs) were used for HRQoL assessed with the PDQ-39 and for cognitive outcomes synthesized on a standardized scale, whereas the mean difference (MD) was used for TMT-A because all contributing studies reported this outcome using the same metric. For SMD analyses, effect sizes were calculated using post-intervention (final-value) group means and the pooled post-intervention within-study standard deviation of the experimental and control groups. Standardized effects were expressed as Hedges’ adjusted g, as implemented in RevMan 5.4, thereby applying the corresponding small-sample bias correction. Post-intervention means, standard deviations (SDs), and sample sizes were extracted directly from the included studies. Baseline SDs and change-score SDs were not used for standardization, and no SD imputation or conversion was performed. Statistical significance was established *a priori* at *p* < 0.05 (Verhagen et al., 1998). Pooled estimates were synthesized using an inverse-variance fixed-effect model. Meta-analyses were conducted separately for each outcome, combining studies that provided sufficiently comparable data for the same measure. Statistical heterogeneity was assessed using Cochran’s Q and quantified using I^2^, interpreted as low (<25%), moderate (25–50%), or high (>50%) (Higgins and Green, 2008). Given the small number of studies contributing to some analyses, heterogeneity estimates were interpreted cautiously and considered together with the magnitude and precision of the pooled effects (Davey et al., 2011). Formal assessment of small-study effects or publication bias using funnel plots or statistical tests for funnel plot asymmetry was not performed because fewer than 10 studies contributed to each meta-analysis. Under these conditions, such methods have limited statistical power and may yield unreliable interpretations (Higgins et al., 2023). Consequently, publication bias could not be formally assessed, and its presence cannot be excluded.

### 2.8 Certainty of evidence

The certainty of evidence was assessed using the Grading of Recommendations Assessment, Development and Evaluation (GRADE) framework (Schünemann et al., 2024) and classified as high, moderate, low, or very low. The included studies were all randomized controlled trials, leading to an initial high rating of evidence certainty. However, this rating was downgraded due to serious concerns identified in one or more GRADE domains, such as risk of bias, inconsistency, indirectness, imprecision, and publication bias. Four reviewers independently assessed the certainty of evidence for each outcome and remained blinded to one another’s judgments until completion. Discrepancies were resolved through consensus, and the final certainty ratings were independently cross-checked by two additional reviewers.

## 3 Results

### 3.1 Selection of studies

A total of 3,037 records were identified through the database search: PubMed 784, Scopus 987, Web of Science 387, and EBSCOhost 879. After removing 27 duplicate records, 3,010 records were screened by title and abstract. During this stage, 2,928 records were excluded, including 1,967 based on title and 961 based on abstract. A total of 82 reports were sought for retrieval, all of which were retrieved and assessed for eligibility. Following full-text assessment, 72 reports were excluded: 32 due to ineligible population, 23 due to ineligible intervention, and 17 due to ineligible study design. Finally, 10 studies were included in the systematic review (Barboza et al., 2019; Fernandes et al., 2015; Jäggi et al., 2023; Meng et al., 2024; San Martín Valenzuela et al., 2020; Schaible et al., 2021; Vergara-Diaz et al., 2018; Wong et al., 2024a,b; Zhou et al., 2024), of which 9 were included in the meta-analysis (Barboza et al., 2019; Fernandes et al., 2015; Jäggi et al., 2023; Meng et al., 2024; San Martín Valenzuela et al., 2020; Vergara-Diaz et al., 2018; Wong et al., 2024a,b; Zhou et al., 2024) (Figure 1).

**FIGURE 1:**
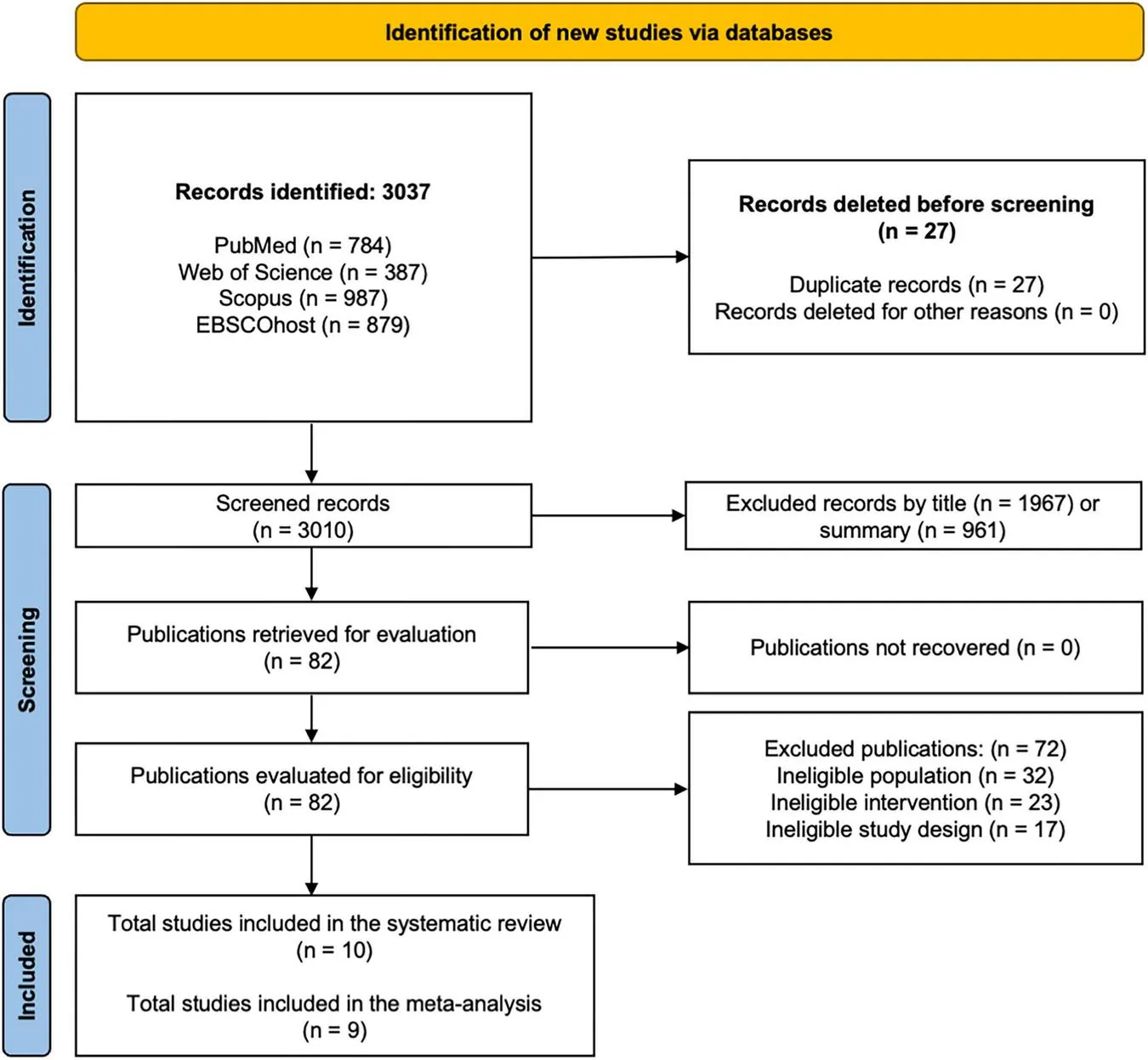
PRISMA 2020 flow diagram of study identification, screening, eligibility assessment, and inclusion in the systematic review and meta-analysis.

### 3.2 Methodological quality

All included studies were randomized controlled trials and were classified as level 1b evidence according to the Oxford Centre for Evidence-Based Medicine framework. However, methodological appraisal identified limitations across several trials, as reflected in the RoB 2 assessment, with studies ranging from low risk of bias to some concerns or high risk of bias.

### 3.3 Risk of bias

The RoB 2 assessment identified important methodological limitations across the included trials. Three studies exhibited a low risk of bias (San Martín Valenzuela et al., 2020; Vergara-Diaz et al., 2018; Wong et al., 2024a), while five studies raised some concerns about risk of bias (Jäggi et al., 2023; Meng et al., 2024; San Martín Valenzuela et al., 2020; Schaible et al., 2021; Vergara-Diaz et al., 2018). Two studies were identified as having a high risk of bias (Barboza et al., 2019; Fernandes et al., 2015). Although the evidence is based exclusively on randomized controlled trials, the interpretation of the pooled findings should be cautious, particularly because most studies presented at least some methodological concerns (Figures 2, 3).

**FIGURE 2:**
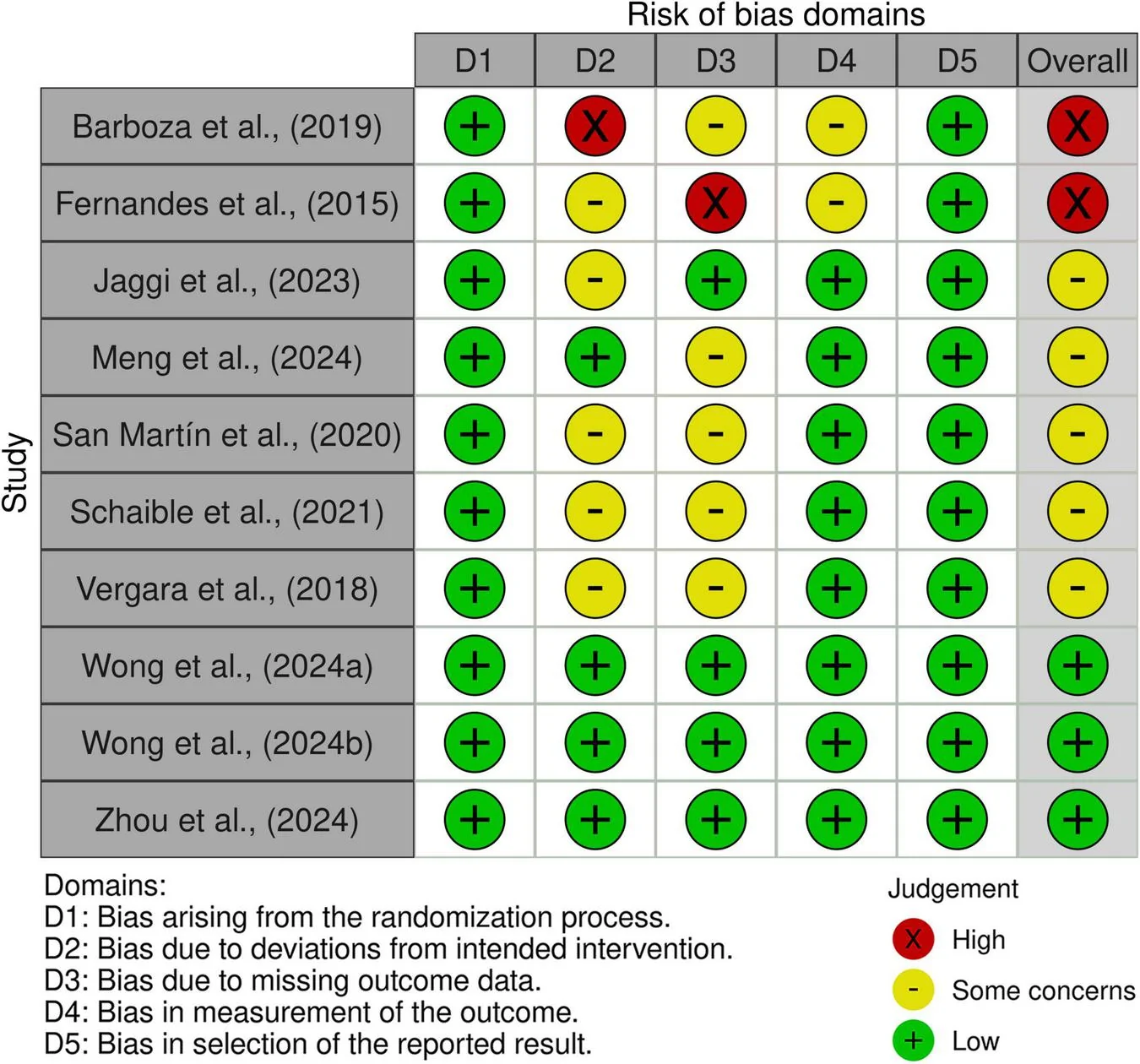
Summary of risk-of-bias judgments for the included randomized controlled trials according to the Cochrane Risk of Bias 2 (RoB 2) tool.

**FIGURE 3:**
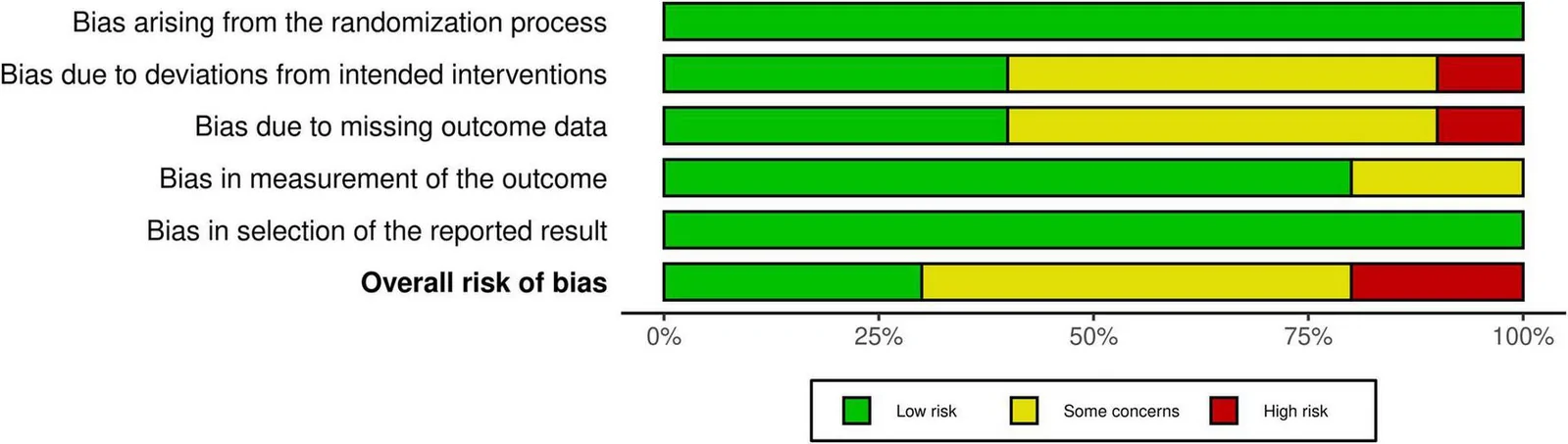
Traffic-light plot of risk-of-bias judgments for individual included randomized controlled trials according to the Cochrane Risk of Bias 2 (RoB 2) tool.

### 3.4 Characteristics of the included studies

A total of ten RCTs were included in this systematic review, of which nine contributed data to the meta-analysis (Barboza et al., 2019; Fernandes et al., 2015; Jäggi et al., 2023; Meng et al., 2024; San Martín Valenzuela et al., 2020; Schaible et al., 2021; Vergara-Diaz et al., 2018; Wong et al., 2024a,b; Zhou et al., 2024). The studies were conducted in Brazil, Portugal, Sweden, Taiwan, China, Spain, Germany, and the United States, with a pooled sample of 453 individuals with PD. Three studies included middle-aged adults, whereas the remaining studies focused mainly on older adults, with a weighted mean age of 64.5 years (Table 2).

**TABLE 2:** Characteristics of the included studies evaluating dual-task interventions on cognitive function and health-related quality of life in people with Parkinson’s disease.

Study	Country	Study design	Initial sample	Groups (n)	Average age (years)	Type of intervention	Training volume			HRQoL assessment	Assessment of cognitive function	Main results
							Week	Frequency (sessions/ weeks)	Session duration (min)			
Barboza et al., 2019	BR	RCT	PD	EG: 29 CG: 29	65.7	Physiotherapy with training cognitive vs. Physiotherapy alone	16	2	90	PDQ	TMT-A and TMT-B	PDQ (*p =* 0.37) TMT-A (*p =* 0.13) TMT-B (*p =* 0.89)
Fernandes et al., 2015	PT	RCT	PD	EG: 7 CG: 8	57	Dual task balance training vs. Single-task training	6	2	60	NR	TMT-A and TMT-B	TMT-A (*p =* 0.950) TMT-B (*p =* 0.345)
Jäggi et al., 2023	SE	RCT	PD	EG: 21 CG: 21	71	Cognitive motor exergame training vs. Traditional Rehabilitation	2 - 4	5	30	NR	TMT-A and TMT-B	PDQ-39 (*p =* 0.172) TMT-A (*p =* 0.135) TMT-B (*p =* 0.024)
Meng et al., 2024	CN	RCT	PD	EG: 14 CG: 14	65	Experimental DTC vs. control group	12	2	–	PDQ-39	MMSE and MoCA	PDQ-39 (*p =* 0.011) MMSE (*p =* 0.198) MoCA (*p =* 0.262)
Schaible et al., 2021	DE	RCT	PD	CG: 12 EG: 14 IG: 15	67	Dual-task group training vs. Single-task training	EG and IG: 4 CG: 8	EG and IG: 4 CG: 2	60	PDQ-39	MMSE	PDQ-39 (*p =* 0.694) MMSE (*p =* 0.591)
San Martín Valenzuela et al., 2020	ES	RCT	PD	EG: 23 CG: 17	67	DT vs. Single-task training	8	2	60	PDQ-39	TMT-A and TMT-B	PDQ-39 (*p =* 0.01) TMT-A (*p =* 0.05) TMT-B (*p =* 0.05)
Vergara-Diaz et al., 2018	US	RCT	PD	EG: 16 CG: 16	57.5	Tai Chi vs. Routine Care	24	2	60	PDQ-39	TMT-A and TMT-B	PDQ-39 (*p =* 0.82) TMT-A (*p =* 0.295) TMT-B (*p =* 0.27)
Wong et al., 2024a	TW	RCT	PD	EG:17 CG: 17	66.5	Motor and cognitive complex training vs. Control training condition	5	3	50	PDQ-39	TMT-A and TMT-B	PDQ-39 (*p =* 0.566) TMT-A (*p =* 0.431) TMT-B (*p =* 0.822)
Wong et al., 2024b	TW	RCT	PD	EG: 11 CG: 10 ECMG: 11	66.14	DLPFC tDCS followed by treadmill- cognitive training vs. Sham/control tDCS followed by treadmill training	6	2	40	NR	MoCA	MoCA (*p* > 0.05)
Zhou et al., 2024	TW	RCT	PD	EG: 14 CG: 14		Dual-task vs. single-task treadmill training	8	2	60	PDQ-39	TMT-A, TMT-B, and MoCA	PDQ-39 (*p =* 0.003) TMT-A (*p =* 0.057) TMT-B (*p =* 0.108) MoCA (*p =* 0.001)

AVLT: Auditory Verbal Learning Test; BR: Brazil; CG: Control Group; CN: China; DE: Germany; D-KEFS: Delis–Kaplan Executive Function System; DLPFC: Dorsolateral Prefrontal Cortex; DST: Digit Span Test; DT: Dual Task; DTC: Dual-Task Cluster; ECMG: Experimental Complex Group; EG: Experimental Group; ES: Spain; FAB: Frontal Assessment Battery; HRQoL: Health-Related Quality of Life; IG: Intensive Group; LSVT BIG: Lee Silverman Voice Treatment – BIG; MMSE: Mini-Mental State Examination; MoCA: Montreal Cognitive Assessment; NR: Not Reported; PANDA: Parkinson Neuropsychometric Dementia Assessment; PD: Parkinson’s Disease; PDQ / PDQ-39: Parkinson’s Disease Questionnaire / 39-item version; PT: Portugal; RCT: Randomized Controlled Trial; ROCF: Rey–Osterrieth Complex Figure; RSCardsT: Reaction Speed Cards Test; SCWT: Stroop Color and Word Test; SE: Sweden; tDCS: Transcranial Direct Current Stimulation; TMT-A / TMT-B: Trail Making Test Parts A and B; TT: Treadmill Training; TW: Taiwan; US: United States; VFT: Verbal Fluency Test; VR: Virtual Reality.

The included interventions showed substantial clinical heterogeneity in content and delivery. Conventional cognitive–motor dual-task training was evaluated in several studies, including balance, gait, treadmill, and group-based protocols (Fernandes et al., 2015; San Martín Valenzuela et al., 2020; Schaible et al., 2021; Zhou et al., 2024). Other studies examined physiotherapy combined with cognitive training (Barboza et al., 2019), cognitive–motor exergames or technology-supported training (Jäggi et al., 2023), dual-task cluster training (Meng et al., 2024), Tai Chi (Vergara-Diaz et al., 2018), and motor–cognitive complex training or treadmill-based protocols combined with tDCS (Wong et al., 2024a,b). Accordingly, the interventions differed in the type of motor activity, cognitive demand, training format, technological support, and degree of integration between motor and cognitive components.

Training dose also varied across studies. Sample sizes in the experimental groups ranged from 7 (Fernandes et al., 2015) to 41 participants (San Martín Valenzuela et al., 2020), intervention duration ranged from 2 (Jäggi et al., 2023) to 24 weeks (Vergara-Diaz et al., 2018), training frequency ranged from two to five sessions per week, and session duration ranged from 30 (Jäggi et al., 2023) to 90 min (Barboza et al., 2019).

### 3.5 Health-related quality of life

The pooled analysis showed a small statistically significant effect in favor of the DT intervention compared with the control condition for HRQoL, as assessed using the PDQ-39 (SMD = 0.35; 95% CI: 0.03–0.67; Z = 2.13; *p =* 0.03), based on a total sample of 157 participants (80 in the DT group and 77 in the control group).

Statistical heterogeneity was low to moderate (I^2^ = 27%; χ^2^ = 5.45; df = 4; *p =* 0.24). Given the small number of studies contributing to this analysis, the heterogeneity estimate should be interpreted cautiously and does not exclude clinically relevant differences between interventions (Figure 4).

**FIGURE 4:**
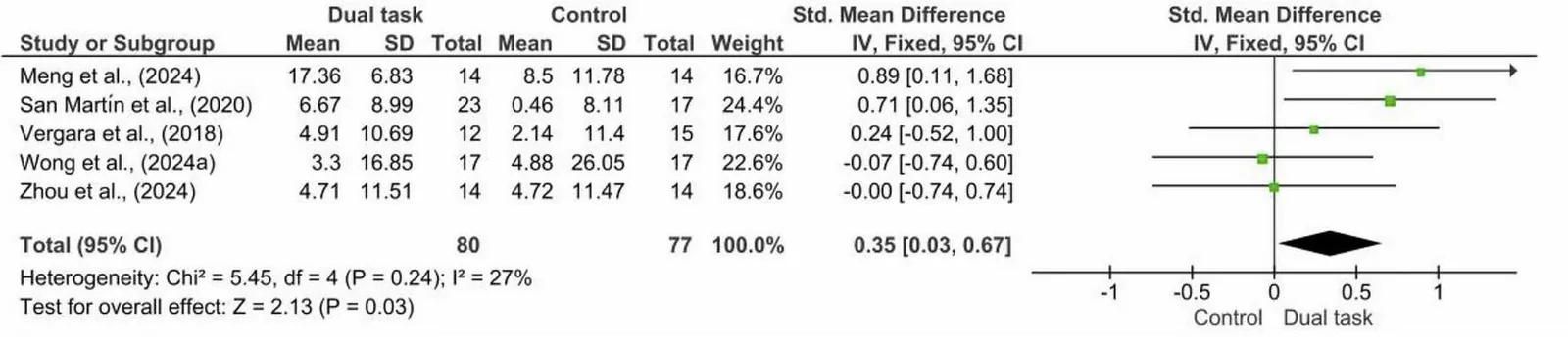
Forest plot of the effect of dual-task interventions on health-related quality of life assessed using the PDQ-39. Effect sizes are presented as standardized mean differences (SMDs) with 95% confidence intervals (CIs) and pooled using an inverse-variance fixed-effect model. The area of each square is proportional to the statistical weight assigned to each study.

### 3.6 Cognitive function

Meta-analyses of cognitive performance assessed using the Trail Making Test Part A (TMT-A), Trail Making Test Part B (TMT-B), and Montreal Cognitive Assessment (MoCA) showed not statistically significant between-group effects. For TMT-A, the pooled analysis of seven studies showed no significant difference between DT interventions and control conditions (MD = 0.99; 95% CI: -3.38 to 5.37; *p =* 0.66), with no observed statistical heterogeneity (I^2^ = 0%).

Similarly, the pooled analysis of seven studies using TMT-B showed no significant between-group effect (SMD = 0.02; 95% CI: -0.24 to 0.29; *p =* 0.86; I^2^ = 0%). Global cognitive function assessed using the MoCA, based on three studies, also showed no significant pooled effect (SMD = 0.19; 95% CI: -0.26 to 0.64; *p =* 0.41; I^2^ = 0%). Given the limited number of studies contributing to these analyses, I^2^ estimates of 0% should not be interpreted as evidence of complete homogeneity between studies (Figure 5).

**FIGURE 5:**
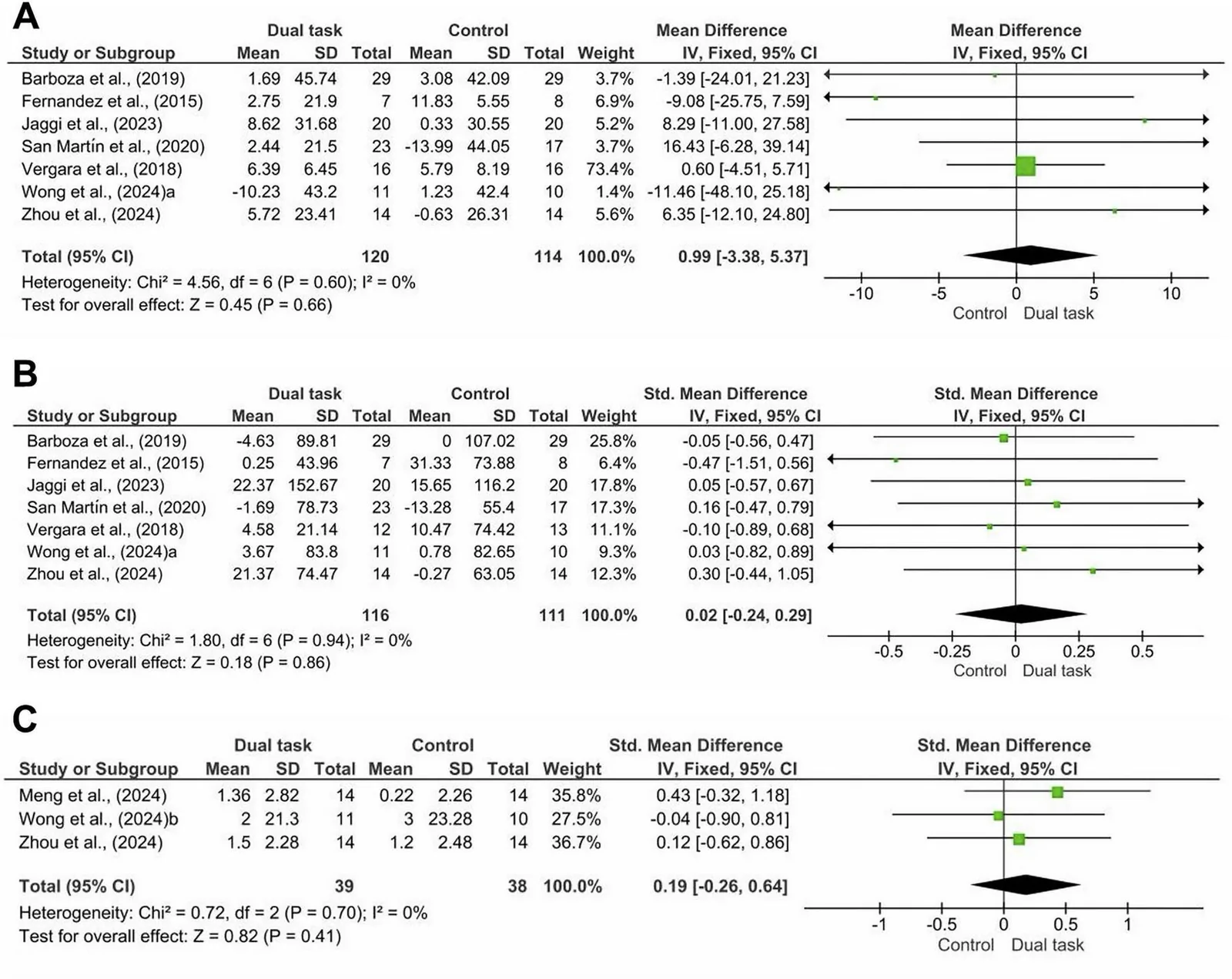
Forest plots of the effect of dual-task interventions on cognitive outcomes: **(A)** Trail Making Test Part A (TMT-A), **(B)** Trail Making Test Part B (TMT-B), and **(C)** Montreal Cognitive Assessment (MoCA). For TMT-A, effects are presented as mean differences (MDs), whereas TMT-B and MoCA are presented as standardized mean differences (SMDs), all with 95% confidence intervals (CIs) and pooled using an inverse-variance fixed-effect model. The area of each square is proportional to the statistical weight assigned to each study.

### 3.7 Certainty of the evidence

The certainty of the evidence was rated as low for the two outcome domains evaluated using GRADE: HRQoL and cognitive function. The cognitive domain comprised the separate meta-analyses of TMT-A, TMT-B, and MoCA. Evidence from randomized controlled trials was downgraded one level because of serious concerns related to risk of bias and one additional level because of serious imprecision, mainly due to the limited information size and the width of the confidence intervals. Accordingly, the pooled estimates should be interpreted cautiously, as future well-designed trials may modify the current conclusions (Table 3).

**TABLE 3:** Certainty of evidence according to the GRADE framework.

Certainty assessment							Number of patients		Effect		Certainty	Importance
Number of studies	Study design	Risk of bias	Inconsistency	Indirect evidence	Imprecision	Other considerations	[Intervention]	[Comparison]	Relative (95% CI)	Absolute (95% CI)		
Health-related quality of life												
5	RCT	Serious^a^	Not serious	Not serious	Serious	None	80/157 (51.0%)	77/157 (49.0%)	Not estimable		Low	IMPORTANT
Cognitive function												
8	RCT	Serious^a^	Not serious	Not serious	Serious	None	120/234 (51.3%)	113/234 (48.7%)	Not estimable		Low	IMPORTANT

CI: confidence interval; GRADE: Grading of Recommendations Assessment, Development and Evaluation; RCT: randomized controlled trial.

^a^Downgraded one level because of concerns related to risk of bias.

### 3.8 Adverse effects and adherence

Adherence was generally high among studies that reported attendance or completion of training sessions, although reporting methods were not consistent across trials. No intervention-related adverse events were reported in the included studies; however, adverse-event monitoring and reporting procedures were not consistently described. Therefore, the absence of reported adverse events should not be interpreted as definitive evidence of intervention safety.

## 4 Discussion

### 4.1 Health-related quality of life

The present meta-analysis demonstrated statistically significant improvements in HRQoL following DT interventions, as assessed using the PDQ-39. The HRQoL findings should also be considered in relation to the measurement properties of the PDQ-39. Although the PDQ-39 is a well-established disease-specific instrument with good evidence of validity and reliability in people with PD, its responsiveness to change varies across domains and has been less consistently demonstrated than other psychometric properties (Brown et al., 2009; Marinus et al., 2002). Therefore, the modest pooled effect observed in the present analysis may partly depend on the ability of the instrument to detect relatively small intervention-related changes. Accordingly, the statistically significant HRQoL effect should be interpreted cautiously with respect to its magnitude and clinical relevance. A crossover trial involving 86 participants, in which the Agility Boot Camp with Cognitive Challenge program, an exercise-based intervention integrating concurrent cognitive tasks, significantly improved HRQoL measured with the PDQ-39 (*p =* 0.003) (Jung et al., 2020). In contrast, a recent systematic review with meta-analysis of adapted boxing exercise in older adults with PD reported no significant improvement in HRQoL (*p =* 0.98), although this intervention was not specifically delivered as a dual cognitive–motor task (Hernandez-Martinez et al., 2025). These findings suggest that the concurrent cognitive component of DT interventions may be relevant for HRQoL benefits, although this interpretation should be considered cautiously. The variability in DT activities across studies may also help explain the observed findings. Interventions that include functional motor tasks combined with attentional or executive demands may be more likely to influence perceived autonomy, confidence, and participation in daily life, whereas protocols with a weaker or less explicit cognitive-motor component may produce less consistent effects. Therefore, the type, specificity, and degree of integration of the dual-task activities should be considered when interpreting the HRQoL results.

### 4.2 Cognitive function

This meta-analysis revealed no statistically significant improvements in cognitive performance following DT interventions, as assessed by TMT-A, TMT-B, and MoCA. These findings should also be interpreted in relation to training specificity and transfer. Motor–cognitive training in people with PD has shown benefits for trained or closely related dual-task outcomes, including gait performance under concurrent cognitive demands and dual-task cost (DTC) (Johansson et al., 2023). DTC quantifies the relative change in performance between single-task and dual-task conditions and can be calculated for either the motor or cognitive component of a dual task, thereby reflecting the degree of interference associated with performing the two tasks concurrently (Johansson et al., 2023). In contrast, TMT-A, TMT-B, and MoCA were not themselves the tasks practiced during most interventions and assess broader cognitive constructs, including visual attention and processing speed, executive control and cognitive flexibility, and global cognition, respectively. Consequently, the absence of significant pooled effects on these measures may partly reflect limited transfer between the specific cognitive–motor demands practiced during training and the standardized cognitive outcomes used to assess treatment effects. This interpretation is consistent with the broader cognitive-training literature, in which improvements are generally more robust for trained or closely related tasks (near transfer) than for more dissimilar, untrained outcomes (far transfer) (Sala et al., 2019). Future trials should therefore assess both task-specific dual-task outcomes and standardized cognitive measures selected according to the cognitive processes explicitly targeted during training.

Although the pooled analyses in the present review did not demonstrate significant effects on standardized cognitive outcomes, some previous studies have reported improvements under specific conditions that may reflect transfer beyond the directly trained dual-task condition. One systematic review reported significant improvement in TMT-A performance in the DT group at 2- and 3-month follow-up, although no significant changes were observed for TMT-B (Wong et al., 2023). Similarly, an RCT evaluating a single 30-min aerobic DT session reported immediate and sustained improvements in attention and processing speed measured with the Symbol Digit Modalities Test (Mollinedo-Cardalda et al., 2025), while a small-scale RCT found that TMT-based training significantly reduced completion times in a global dual-task condition (Silva et al., 2021). These findings indicate that transfer to standardized cognitive outcomes may occur under specific intervention conditions; however, the available evidence remains inconsistent and does not currently support a reliable far-transfer effect.

The absence of significant pooled cognitive effects in the present review may reflect variability in intervention content, cognitive task specificity, outcome sensitivity, and participant characteristics. Some protocols may have emphasized motor or functional performance with an added cognitive component, whereas others more explicitly targeted cognitive–motor integration. These differences may influence the extent to which training effects transfer to standardized cognitive outcomes. Overall, the literature remains heterogeneous, with some studies reporting improvements in specific domains such as attention and processing speed, whereas others show no significant cognitive effects (Monaghan et al., 2023). Future studies should use sensitive, domain-specific cognitive outcomes alongside task-specific dual-task measures and should consider disease stage and baseline cognitive status when examining the short- and long-term cognitive effects of DT interventions in PD (Bloem et al., 2021).

### 4.3 Limitations and strengths

This review has several limitations. The meta-analysis included a limited number of cognitive and HRQoL outcomes, and some studies did not provide comparable post-intervention data. Variability in sample size, intervention dose, training characteristics, and control conditions also contributed to clinical heterogeneity and limited the identification of the most effective DT components. In addition, the literature search was restricted to PubMed, EBSCOhost, Scopus, and Web of Science and did not include other potentially relevant databases such as Embase, CENTRAL, PsycINFO, or PEDro; therefore, some eligible studies may have been missed. The small number of studies contributing to each meta-analysis also precluded a reliable formal assessment of publication bias. Although study selection, data extraction, risk-of-bias assessment, and certainty-of-evidence assessment were conducted independently by multiple reviewers, complete blinding to the content of full-text reports was not possible, and some degree of reviewer judgment was therefore unavoidable.

Despite these limitations, all included studies were randomized controlled trials, and the review procedures incorporated independent assessment, consensus resolution, and additional cross-checking. The use of validated outcome measures facilitated comparability across studies. Adherence was generally high among studies that reported attendance or completion of training sessions; however, reporting methods were not consistent across trials. Similarly, no intervention-related adverse events were reported, but adverse-event monitoring and reporting procedures were not consistently described. Therefore, the absence of reported adverse events should not be interpreted as definitive evidence of intervention safety.

### 4.4 Practical applications

This systematic review and meta-analysis found a small statistically significant improvement in HRQoL following DT interventions, whereas the pooled evidence did not demonstrate significant effects on the cognitive outcomes assessed. DT interventions may be executed via brief, intensive training protocols or extended programs with reduced session frequency, providing flexibility to meet specific patient requirements and diverse clinical circumstances. This adaptability enables the modification of training intensity according to institutional resources and participant attributes.

The present findings do not support conclusions regarding the superiority of specific DT components, delivery models, or training environments. The included interventions were delivered through heterogeneous formats, including gait- and balance-based exercises, treadmill training, group-based programs, Tai Chi, cognitive–motor exergames, and technology-supported approaches. These differences may influence task demands, progression, supervision, and transfer to everyday functioning, but the limited number of studies precludes meaningful comparisons between training formats or environments. The current evidence does not establish whether individually delivered, group-based, or other DT delivery formats are more effective. Therefore, the choice of delivery format should be guided by clinical feasibility and available resources until comparative trials determine whether specific approaches provide greater benefits. Future trials should provide detailed descriptions of the motor and cognitive components, progression criteria, training setting, intervention dose, and delivery format to clarify whether these characteristics modify treatment effects.

### 4.5 Clinical applications

In clinical practice, DT interventions may be considered as one component of rehabilitation for people with PD, but the current evidence does not identify a clearly superior training format. Accordingly, no specific delivery model can presently be recommended over another. Where appropriate, motor tasks may be combined with progressively challenging cognitive demands; however, the optimal type, intensity, progression, and delivery format remain uncertain and require further investigation.

Technology-supported approaches were included in some trials, such as cognitive–motor exergames and adjunctive neuromodulation combined with motor training (Davey et al., 2011; Schaible et al., 2021). However, the small number of studies and substantial variability in intervention content preclude conclusions regarding whether technology-supported DT formats are superior to conventional approaches. Future trials should directly compare delivery formats and clearly report the cognitive and motor components, progression criteria, and training dose.

### 4.6 Epidemiological applications

From a public health perspective, DT interventions may represent a potentially useful component of rehabilitation for people with PD, particularly in relation to HRQoL. However, the statistically significant HRQoL effect was small, based on a limited number of studies, and supported by low-certainty evidence; therefore, it is not sufficient on its own to justify broad implementation. In contrast, the pooled analyses did not demonstrate significant benefits for cognitive outcomes, so the current evidence does not support implementation of DT specifically for cognitive improvement. Larger and methodologically robust RCTs are therefore needed for different reasons across these outcome domains: to determine the clinical relevance and reproducibility of the HRQoL effect and to establish whether cognitive benefits can be demonstrated under more consistent and adequately powered intervention conditions. Future studies should also examine whether intervention effects differ according to age, disease stage, and baseline cognitive status, and should evaluate feasibility, resource requirements, and longer-term outcomes in routine clinical and community settings.

## 5 Conclusion

Dual-task interventions were associated with a statistically significant but modest improvement in HRQoL, as measured by the PDQ-39, whereas no significant between-group effects were observed for cognitive outcomes assessed with TMT-A, TMT-B, or MoCA. These findings should be interpreted cautiously given the small number of studies, methodological limitations, and clinical heterogeneity across interventions. Although adherence was generally high and no intervention-related adverse events were reported, the available evidence does not allow firm conclusions regarding the safety or superiority of DT over conventional rehabilitation.

Future RCTs should investigate whether incorporating ecologically relevant functional tasks into DT training, together with outcome measures that capture transfer to everyday functioning, improves the clinical relevance and generalizability of intervention effects.

## Data Availability

The original contributions presented in the study are included in the article/supplementary material, further inquiries can be directed to the corresponding authors.
